# Mechanism of Huoluo Xiaoling Dan in the Treatment of Psoriasis Based on Network Pharmacology and Molecular Docking

**DOI:** 10.1155/2022/7053613

**Published:** 2022-02-27

**Authors:** Ke Gong, Wen Guo, Kaiqing Du, Fang Wang, Mengli Li, Jianhui Guo

**Affiliations:** ^1^Hebei Province Cangzhou Hospital of Integrated Traditional and Western Medicine, Cangzhou, China; ^2^Department of Traditional Chinese Medicine Surgery, Hebei University of Chinese Medicine, Shijiazhuang, China; ^3^Traditional Chinese Medicine Hospital of Jiaozuo City, Henan Province, Jiaozuo, China

## Abstract

**Objective:**

To explore the mechanism of the action of Huoluo Xiaoling Dan (HLXLD) in the treatment of psoriasis based on network pharmacology and molecular docking.

**Methods:**

The main active components and targets of HLXLD were collected from CMSP, and the targets related to psoriasis were collected from GeneCards, OMIM, TTD, DisGeNET, and DrugBank. Drug disease target genes were obtained by Venny tools, drug-component-target networks were constructed and analyzed, and pathway enrichment analysis was performed. AutoDockTools is used to connect the core components and the target, and PyMOL software is used to visualize the results.

**Results:**

126 active components (such as quercetin, luteolin, tanshinone IIA, dihydrotanshinlactone, and beta-sitosterol) and 238 targets of HLXLD were screened out. 1,293 targets of psoriasis were obtained, and 123 drug-disease targets were identified. Key targets included AKT1, TNF, IL6, TP53, VEGFA, JUN, CASP3, IL1B, STAT3, PTGS2, HIF1A, EGF, MYC, EGFR, MMP9, and PPARG. Enrichment analysis showed that 735 GO analysis and 85 KEGG pathways were mainly involved in biological processes such as response to the drug, inflammatory response, gene expression, and cell proliferation and apoptosis, as well as signal pathways such as cancer, TNF, HIF-1, and T cell receptor. Molecular docking showed that there was strong binding activity between the active ingredient and the target protein.

**Conclusions:**

HLXLD could treat psoriasis through multicomponents, multitargets, and multipathways, which provides a new theoretical basis for further basic research and clinical application.

## 1. Introduction

Psoriasis was a common inflammatory reactive skin disease with erythema and scaly lesions as the main manifestations, accompanied by varying degrees of pruritus, with a chronic course and easy recurrence [1]. The pathogenesis of the disease was not completely clear, and the etiology was complex, which was related to genetic, environmental, immune, and other factors [2]. Syndrome differentiation and treatment was a unique advantage of traditional Chinese medicine (TCM).

TCM treatment of psoriasis is mostly based on blood, and blood stasis syndrome is one of the most common syndromes [3]. In recent years, many doctors had treated psoriasis from the perspective of collateral disease theory. Based on the Huoluo Xiaoling Dan (HLXLD) composed of Radix Salviae (Danshen (DS)), Angelica Sinensis (Danggui (DG)), Frankincense (Ru Xiang(RS)), and Myrrha (Moyao (MY)), they have added and subtracted according to the syndrome and achieved good results [4, 5], but the molecular mechanism of the treatment has not been clear.

Therefore, this study through network pharmacology to predict the potential targets and signal pathways of HLXLD in the treatment of psoriasis and combines it with molecular docking technology to assist verification, to provide a certain theoretical basis for the in-depth study of HLXLD in the treatment of psoriasis.

## 2. Materials and Methods

### 2.1. Screening of Active Components and Targets of HLXLD

On the Traditional Chinese Medicine Database and Analysis Platform [6] (TCMSP, https://www.tcmspw.com/), the names of four Chinese herbal medicines were input in succession to obtain the corresponding chemical compounds and related information. According to the principle of pharmacokinetics (ADME), the oral bioavailability (OB) ≥ 30% and drug-likeness (DL) ≥0.18 were set as the screening conditions to obtain the effective components and corresponding targets of each drug of HLXLD. The Uniprot database [7] (http://www.uniprot.org/) was used to screen the target genes from human attributes and was verified, and the target names were standardized into official symbols.

### 2.2. Collection of Target Proteins Associated with Psoriasis

To ensure the comprehensiveness and accuracy of the data, the potential targets of psoriasis in GeneCards [8] (https://www.genecards.org/), OMIM [9] (https://omim.org/), TTD [10] (http://db.idrblab.net/ttd/), DisGeNET [11] (https://www.disgenet.org/), and Drugbank [12] (https://go.drugbank.com/) were searched with “psoriasis” as keywords. In the GeneCards database, the score indicates the closeness between the target and the disease, so the target with a score greater than the median was set as the potential target of psoriasis. The targets obtained from the above disease database were combined, and the repetition was removed to get the psoriasis-related targets, and the Uniprot database is used to standardize the target names through the above methods.

### 2.3. Identification of Potential Therapeutic Targets of HLXLD in the Treatment of Psoriasis

The two target sets were input, respectively, to the Venny 2.1.0 to acquire the common targets, and the Venn diagram was drawn to obtain the potential targets of HLXLD in the treatment of psoriasis.

### 2.4. Construction of the Drug-Compound-Target Network

The obtained active components of HLXLD and the potential targets for psoriasis treatment were imported into Cytoscape 3.8.2 [13] software to draw the drug-compound-target network diagram for visual analysis, in which “node” was used to represent drugs, components, or targets, and “edge” was used to represent the relationship between nodes. The network parameters of each node were analyzed based on the network analyzer and cytoNCA plugins.

### 2.5. Construction of Protein-Protein Interaction (PPI) Network Map and Core Targets' Screening

The potential targets screened above were imported into the STRING [14] (https://www.string-db.org/), “Homo sapiens” was selected, the minimum required interaction score was set to ≥ 0.4, the free nodes were hidden, the PPI network was built, and the corresponding files were exported. The downloaded results were uploaded to Cytoscape3.8.2 software for analysis. The network analyzer and cytoNCA plugins were used for topology analysis. The core targets were screened by taking the median of 2 times of degree, and the median of Betweenness Centrality (BC) and Closeness Centrality (CC) as card values.

### 2.6. Gene Ontology (GO) Functional Enrichment and Kyoto Encyclopedia of Genes and Genomes (KEGG) Pathway Analysis

The previously obtained potential targets were imported into the DAVID database [15] (https://david.ncifcrf.gov/), and the species was defined as “Homo sapiens.” The GO function and KEGG pathway enrichment of common target genes of HLXLD and psoriasis were analyzed. The results were visualized by using Bioinformatics online mapping tool (http://www.bioinformatics.com.cn/), and the network diagram of “disease-pathway-target-component drug” was constructed by using Cytoscape 3.8.2.

### 2.7. Molecular Docking

Molecular docking was applied for the key components with the top 5 degree values in the drug-compound-target network and the core targets with the top 5 degree values in the PPI network. The protein structure of the core target was downloaded from the PDB [16] database (https://www.rcsb.org/). The selection criteria [17] are as follows: (1) X-ray structures with a resolution of 2.5 Å or better were included, if available; (2) if two or more structures were available, that with the best solution was selected; (3) a structure with a ligand bound to its nucleotide-binding site was selected; (4) non-modified and non-phosphorylated residues found in the binding site were selected with priority; (5) the organism was human. The small molecular structure of key components was downloaded from the TCMSP database. The PyMOL 2.4.0 [18] software was used to dewater, hydrogenate, and separate the original ligand of the core target protein. The molecular docking was completed in the AutoDockVina [19] software. When the binding energy was negative, it indicated that the receptor and ligand can bind spontaneously; when the binding energy was less than -5 kcal mol^−1^, it indicated that there was good binding activity between the receptor and the ligand [17]. The results with higher activity were visualized by PyMOL 2.4.0.

## 3. Results

### 3.1. Screening of Active Components and Targets of HLXLD

Through TCMSP database retrieval, DS, DG, RX, and MY had 66, 8, 9, and 45 active ingredients, respectively. A total of 126 active components of HLXLD were obtained after merging and removing duplicates, and the corresponding target proteins were further obtained. After being transformed into standard gene names in the UniProt database and removing duplicates, a total of 238 predicted targets were obtained.

### 3.2. Collection of Target Proteins Associated with Psoriasis

**Figure 1:**
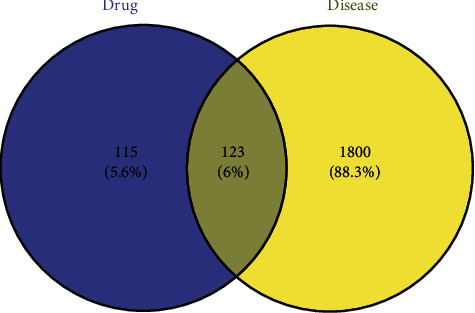
Venn diagram of intersecting targets of HLXLD and psoriasis.

After merging and removing the duplication of acne disease target genes collected by GeneCards, OMIM, TTD, DisGeNET, and DrugBank databases, a total of 1,923 related target proteins were obtained. After matching the target genes of the active components of HLXLD with the target genes related to psoriasis, the intersection was taken, and 123 common genes were obtained through the Venn diagram (Figure 1).

### 3.3. Construction of the Drug-Compound-Target Network

**Figure 2:**
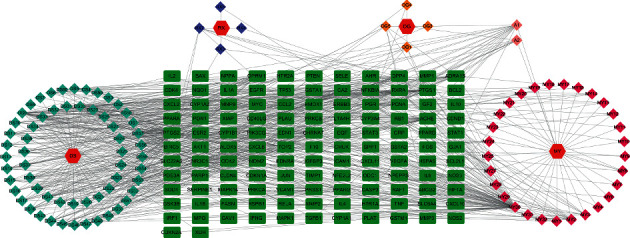
The drug-compound-target network of HLXLD.

The active components and the corresponding targets of HLXLD in the treatment of psoriasis were introduced into the Cytoscape 3.8.2 software to construct the network diagram of the drug-compound-target network which included 225 points and 759 edges (Figure 2). Among them, the red hexagon node represents the drug, the light blue diamond node represents the effective components of DS, the orange diamond node represents the effective components of DG, the dark blue diamond node represents the effective components of RX, the purple diamond node represents the effective components of MY, the pink diamond node represents the common components of DG and MY, and the green square node represents the action target. After network topology analysis, the median of degree, BC, and CC was set as the card values. Among the effective active ingredients screened out, quercetin (MOL000098) had the highest degree value, acting on 93 drug targets. In the descending order, luteolin (MOL000006) acted on 37 drug targets, tanshinone IIA (MOL007154) acted on 22 drug targets, and dihydrotanshinlactone (MOL007100) acted on 18 drug targets, *β*-sitosterol (MOL000358) acted on 17 drug targets.  Table 1 shows that the 27 key drug components were analyzed by topology. Figure 2 and Table 1 show the characteristics of HLXLD in the treatment of psoriasis with multicomponent and multitarget.

### 3.4. PPI Network Construction and Topology Analysis

**Figure 3:**
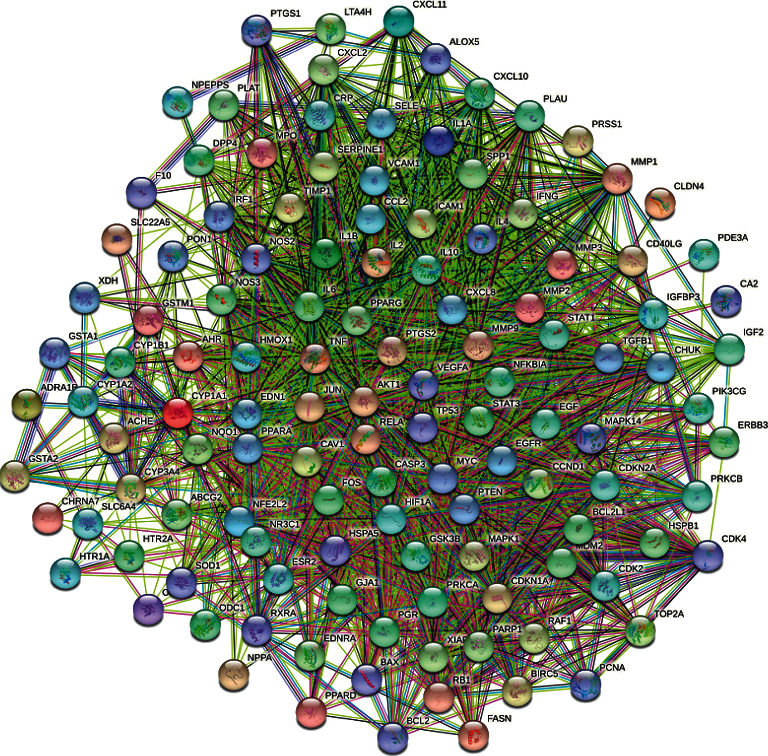
The PPI network of HLXLD in the treatment of psoriasis.

**Figure 4:**
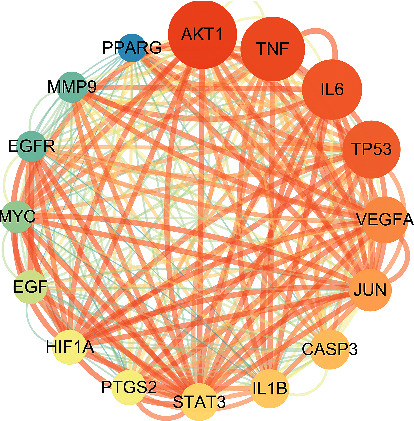
Core target PPI network.

123 intersection targets brought in the STRING database to set up the PPI network (Figure 3). The network consists of 123 points and 2,413 edges, with an average degree of 39.2. Imported into Cytoscape 3.8.2 software, network analyzer and cytoNCA plugins were used to further analyze the PPI network, and 16 targets with degree ≥74, BC ≥ 0.0021906, and CC ≥ 0.57819905 were selected. These targets played a key role in the PPI network and are the core targets of HLXLD in the treatment of psoriasis. The core protein interaction was further visualized (Figure 4). There were 16 nodes and 240 edges in the network. The size and color of nodes reflect the size of the degree. The larger the node, the more red color indicates the higher degree. The thickness of edges reflects the size of the connection score between nodes. The thicker the color indicates the larger the connection score between nodes. The basic information is shown in Table 2.

### 3.5. GO Enrichment Analysis

**Figure 5:**
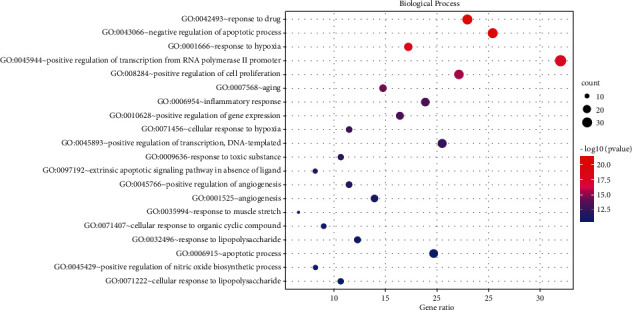
BP enrichment analysis of 123 nodes.

**Figure 6:**
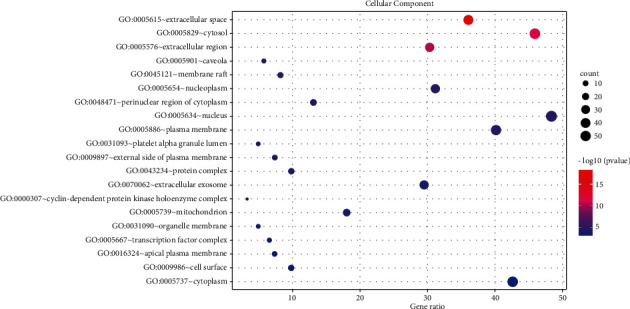
CC enrichment analysis of 123 nodes.

**Figure 7:**
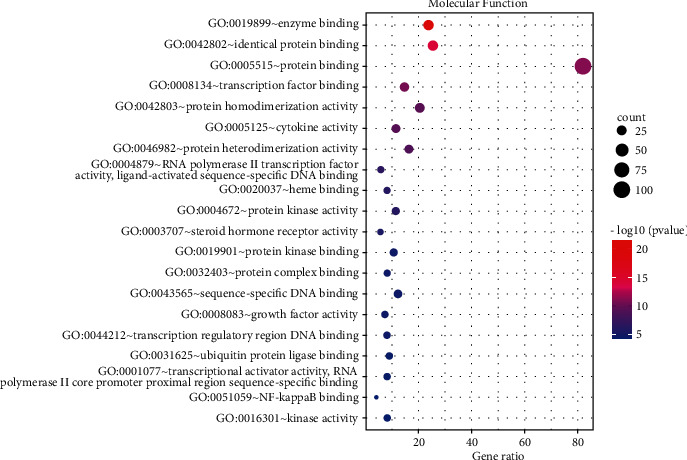
MF enrichment analysis of 123 nodes.

123 common targets were enriched and analyzed by the David database, and a total of 735 were obtained, including 588 biological process (BP), 49 cellular component (CC), and 98 molecular function (MF). The top 20 results were selected according to the *P* value to draw the bubble chart (Figures 5–7). The smaller the *P* value, the more the color of the point tends to red, the more the number of enriched genes, and the larger the area of the point.

### 3.6. KEGG Pathway Enrichment Analysis

**Figure 8:**
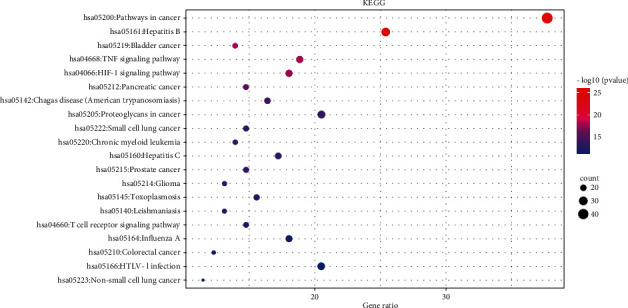
The top 20 pathways of KEGG enrichment.

**Figure 9:**
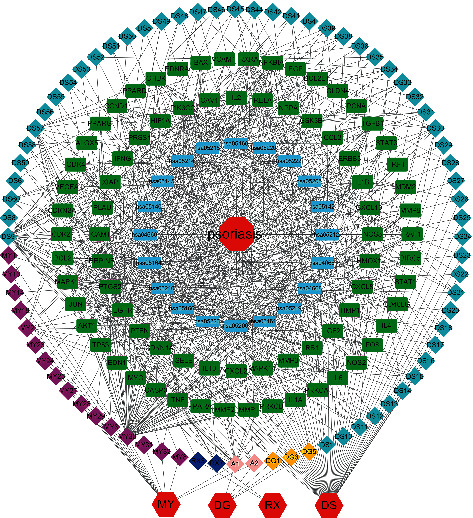
The disease-pathway-target-compound-drug network.

A total of 85 pathways were obtained by KEGG pathway enrichment analysis. The top 20 results were selected according to the *P* value to draw the bubble diagram (Figure 8). The disease-pathway-target-compound-drug network is further constructed through Cytoscape 3.8.2 (Figure 9). The network consists of 179 points (including 1 disease, 20 pathways, 78 targets, 76 components, and 4 drugs) and 844 edges.

### 3.7. Molecular Docking of Main Active Components with Core Targets

**Figure 10:**
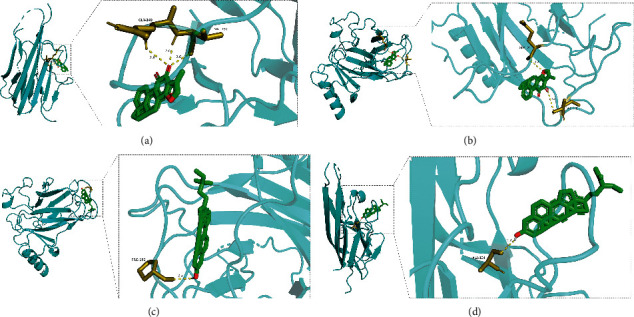
Molecular docking mode. (a) Tanshinone IIA-TNF, (b) beta-sitosterol-TNF, (c) beta-sitosterol-TP53, and (d) tanshinone IIA-TP53.

Quercetin, luteolin, tanshinone IIA, *β*-sitosterol, and dihydrotanshinlactone ranked among the top 5 key components in the drug-compound-target network. The top 5 targets with degree value in the core target PPI network were AKT1, TNF, IL6, TP53, and VEGFA. The above components and targets were docked by the AutoDockVina software. The results are shown in Table 3. The average binding energy was -6.65 kcal mol^−1^, among which tanshinone IIA-TNF, *β*-sitosterol-TNF, *β*-sitosterol-TP53, and tanshinone IIA-TP53 had the highest affinity. The molecular docking results showed that all binding energies were negative, and most of them were less than -5.0 kcal mol^−1^. The binding pattern diagram was drawn by PyMOL 2.4.0 for the docking results with binding energy ≤ -9 kcal mol^−1^ (Figure 10).

## 4. Discussion

The course of psoriasis was prolonged and easy to relapse. The skin lesions of psoriasis were thick and dark, and there were obvious abnormalities in the microcirculation of patients with psoriasis [20, 21]. These clinical characteristics were closely related to the “blood stasis” factor of TCM, which was very consistent with the thought of “long illness entering the collaterals” in the theory of collateral diseases. As a classic prescription for the treatment of collateral diseases, HLXLD was from Integrating Chinese and Western Medicine and was composed of DS, DG, RX, and MY. Danshensu could effectively improve the skin lesions of the psoriasis mouse model induced by imiquimod (IMQ). The higher the concentration, the more obvious the improvement of skin lesions. At the same time, it could also reduce the expression of Yes-related protein (YAP) in skin lesions [22]. YAP was highly expressed in psoriasis and could participate in the pathogenesis by regulating the proliferation and apoptosis of keratinocytes [23]. Salvianolic acid B could improve the skin lesions of IMQ-induced psoriasis mouse models by inhibiting the PI3K/AKT pathway and downregulating the expression of keratin markers [24]. Angelica polysaccharides could downregulate the protein expression of NF-*κ*B in peripheral blood mononuclear cells and reduce the secretion of IFN-*γ* in patients with psoriasis [25]. Mastic acid was also widely used in the clinic because of its strong anti-inflammatory ability [26]. A clinical trial in Italy used boswellic acid to treat psoriasis with an effective rate of up to 70% [27]. Myrrhosterone could significantly inhibit the proliferation of HaCaT cells, induce apoptosis, downregulate the expression of psoriasis-related genes mRNA, and reduce the severity of skin lesions in mice with psoriasis [28]. However, the target and signal pathways of psoriasis treatment from the perspective of “collateral disease theory” have still been unclear. TCM had the characteristics of multicomponent, multitarget, and multichannel in the treatment of diseases. Therefore, combined with the big-data analysis method of network pharmacology, this study aimed to explore the mechanism of HLXLD in the treatment of psoriasis and provide theoretical support for follow-up research.

The results showed that there are 123 potential targets of HLXLD in the treatment of psoriasis, involving 98 active components of HLXLD. According to topological analysis, the main components were quercetin, luteolin, tanshinone IIA, *β*-sitosterol, etc., as shown in Table 1. Previous studies have shown that quercetin and luteolin could inhibit the activation of the NF-*κ*B pathway, reduce the levels of serum inflammatory factors such as TNF-*α*, IL-6, and IL-17, and significantly reduce the PASI score of IMQ-induced psoriasis mouse models [29–31]. In addition, quercetin could significantly reduce the expression level of tyrosine kinase in HaCaT cells [32]. Luteolin could promote the expression of HSP90 in HaCaT cells, reduce the ratio of Th1/Th2 and Th17/Treg in the immune cells of psoriasis mice, and inhibit the increase of Th1 and Th17 in peripheral blood [33]. Tanshinone IIA could inhibit the proliferation of keratinocytes and induce apoptosis in mouse models of psoriasis, thereby reducing the appearance of skin lesions [34, 35]. Another study found that cryptotanshinone could reduce epidermal hyperplasia by inhibiting the activation of STAT3 [36].

The potential target PPI network of HLXLD in the treatment of psoriasis (Figure 3) had selected the core targets by topological analysis (Figure 4), indicating that HLXLD may treat psoriasis by acting on core targets such as AKT1, TNF, IL6, TP53, VEGFA, JUN, CASP3, IL1*β*, STAT3, PTGS2, HIF1A, EGF, MYC, EGFR, MMP9, and PPARG. Many studies have shown that the excessive proliferation of psoriasis keratinocytes was closely related to the increase of AKT1 levels in skin lesions [37, 38]. The dysregulation of the Akt-FOXO1 pathway led to T cell dysfunction, which was also widespread in patients with psoriasis [39]. Inflammation-related factors such as TNF, IL6, and IL1*β* were abnormal throughout the pathogenesis of psoriasis [40]. Growth factors and their receptors such as VEGFA, EGF, and EGFR were also inextricably linked with psoriasis [41–44]. The above point of view had long become a consensus in dermatology. The protein P53 encoded by TP53was called the “Genome Guardian.” During the cell cycle, p53 could not only repair cell cycle arrest in the G1 phase through the expression of p21 but also mediate cell death through the Bcl-2/Bax pathway [45]. Moorchung's study found that p53 was an important protein that regulated the apoptosis process of psoriasis epidermal cells [46]. A clinical study used UV to treat psoriasis. After the course of treatment, it was found that p53 and Foxp3 decreased significantly, and it was speculated that p53 was an essential protein for UV-induced Foxp3 transcription [47]. c-JUN was a pathway closely related to a variety of autoimmune diseases. c-JUN activation could stimulate the production of inflammatory factor IL-6, thereby further aggravating the inflammatory response in psoriasis skin lesions [48]. Bears found that the expression of CASP3 was positively correlated with the condition of psoriasis, especially with the early psoriasis lesions located at the extremities [49]. STAT3 could regulate T cell differentiation. Studies have found that mice with high expression of the STAT3 pathway can spontaneously develop psoriasis-like skin lesions [50]. In the pathogenesis of psoriasis, the STAT3 pathway was involved in regulating the secretion of Th17 cytokines. When the STAT3 pathway was overactive, it could promote the excessive proliferation of keratinocytes and the production of IL6 and IL17. These cytokines, in turn, trigger Th17 and STAT3 signaling pathways, resulting in a sustained inflammatory response [51]. In addition, STAT3 also activated the transcription of related genes by targeting its promoter region, thereby forming a regulatory feedback loop that affected the proliferation and apoptosis of HaCaT cells [52]. Studies have found that HIF1A levels in patients with psoriasis were significantly higher than those in normal patients and were positively correlated with microvessel density in skin lesions [53]. Many other studies have reported that the protein expression levels of PPARG [54], MMP9 [55], and MYC [56] were also strongly correlated with the incidence of psoriasis.

The results of GO functional enrichment analysis and KEGG pathway enrichment showed that the mechanism of HLXLD treatment of psoriasis mainly focuses on drug response, negative regulation of apoptosis process, hypoxia response, positive regulation of RNA polymerase II promoter transcription, and positive regulation of cells. Proliferation, aging, inflammation, positive regulation of gene expression, positive regulation of transcription, DNA templating, etc., and the pathways related to the treatment of psoriasis by HLXLD mainly involved pathways in cancer (including multisystem and multiorgan cancers), TNF signaling pathway, HIF-1 signaling pathway, T cell receptor signaling pathway, etc. At present, more and more pieces of evidence supported the correlation between cancer and psoriasis. Several meta-analyses and retrospective studies [57–60] have found that the risk of cancer in patients with psoriasis was higher than that of normal people, especially skin cancer, lymphatic cancer [58], colorectal cancer [59]^,^ and lung cancer [60], etc. TNF had strong biological activity and played an important role in the pathogenesis of psoriasis. The TNF/IL23/IL17A axis was also widely involved in the body's immune response, promoting the occurrence of psoriasis inflammatory response and the proliferation of epidermal cells [61]. The increase of HIF-1 in keratinocytes helped promote angiogenesis and skin inflammation [62]. Another study found that HIF1A might promote the glycolysis process of psoriasis Vulgaris by increasing the expression of CD147 and GLUT1 [63]. From an immune perspective, psoriasis was a chronic skin disease associated with T cell-mediated inflammation. The activation of T cells promoted the proliferation and migration of keratinocytes, thereby causing or accelerating the progression of the disease. At present, most monoclonal antibody preparations were aimed at this mechanism and block downstream inflammatory mediators such as TNF and IL17A to treat psoriasis [64].

## 5. Conclusion

To sum up, based on the network pharmacology method, this study systematically expounded the relationship between HLXLD in the treatment of psoriasis through multicomponent, multitarget, and multichannel, and verifies the strong binding activity between key components and core targets by molecular docking technology. The results showed that HLXLD might act on AKT1, IL6, TNF, TP53, VEGFA, EGF, EGFR, CXCL8, MMP9, MAPK8, and other targets through active components such as quercetin, luteolin, tanshinone IIA, *β*-Sitosterol, and dihydrodanshenlide, involving multiple signal pathways such as cancer signaling pathway, TNF signaling pathway, HIF-1 signaling pathway, and T cell receptor signaling pathway, participating in the drug reaction, inflammatory reaction, gene expression, cell proliferation, and apoptosis, to play a role in the treatment of psoriasis, which provided a theoretical basis for the further application of HLXLD in the clinic. However, because this study mainly relied on the network database, there were still some limitations. The specific drug action mechanism needs further experimental verification in vivo and in vitro.

## Figures and Tables

**Table 1:** The drug components of HLXLD in the treatment of psoriasis.

MOL ID	Molecule name	OB	DL	Degree	Source
MOL000098	Quercetin	46.43	0.28	96	MY
MOL000006	Luteolin	36.16	0.25	38	DS
MOL007154	Tanshinone IIA	49.89	0.40	23	DS
MOL000358	Beta-sitosterol	36.91	0.75	19	DG、MY
MOL007100	Dihydrotanshinlactone	38.68	0.32	18	DS
MOL007088	Cryptotanshinone	52.34	0.40	16	DS
MOL007093	Danshexinkum D	38.88	0.55	16	DS
MOL007119	Milrinone I	49.68	0.32	14	DS
MOL007108	Isocryptotanshinone	54.98	0.39	14	DS
MOL007124	Neocryptotanshinone II	39.46	0.23	13	DS
MOL007041	2-Isopropyl-8-methylphenanthrene-3,4-dione	40.86	0.23	13	DS
MOL007049	4-Methylenemiltirone	34.35	0.23	13	DS
MOL007098	Deoxyneocryptotanshinone	49.40	0.29	12	DS
MOL007105	Epidanshenspiroketallactone	68.27	0.31	12	DS
MOL001601	1,2,5,6-Tetrahydrotanshinone	38.75	0.36	12	DS
MOL000449	Stigmasterol	43.83	0.76	11	DG、MY
MOL007111	Isotanshinone II	49.92	0.40	11	DS
MOL007094	Danshenspiroketallactone	50.43	0.31	11	DS
MOL007061	Methylenetanshinquinone	37.07	0.36	11	DS
MOL007145	Salviolone	31.72	0.24	11	DS
MOL001004	Pelargonidin	37.99	0.21	10	MY
MOL007122	Miltirone	38.76	0.25	10	DS
MOL007127	1-Methyl-8,9-dihydro-7H-naphtho[5,6-g]benzofuran-6,10,11-trione	34.72	0.37	10	DS
MOL007036	5,6-Dihydroxy-7-isopropyl-1,1-dimethyl-2,3-dihydrophenanthren-4-one	33.77	0.29	9	DS
MOL007125	Neocryptotanshinone	52.49	0.32	10	DS
MOL007059	3-*β*-Hydroxymethyllenetanshiquinone	32.16	0.41	10	DS
MOL007069	Przewaquinone C	55.74	0.40	10	DS

**Table 2:** The core target topological parameters of HLXLD in the treatment of psoriasis.

Gene	Gene name	BC	DC	Degree
AKT1	RAC-alpha serine/threonine-protein kinase	0.06452241	0.82993197	97
TNF	Tumor necrosis factor	0.05075691	0.81879195	95
IL6	Interleukin-6	0.03833700	0.80794702	93
TP53	Cellular tumor antigen p53	0.03368498	0.80263158	92
VEGFA	Vascular endothelial growth factor A	0.01953143	0.77707006	87
JUN	Transcription factor AP-1	0.03194387	0.76250000	85
CASP3	Caspase-3	0.01684584	0.75776398	83
IL1B	Interleukin-1 beta	0.01865965	0.75308642	82
STAT3	Signal transducer and activator of transcription 3	0.01224804	0.74390244	81
PTGS2	Prostaglandin G/H synthase 2	0.01437445	0.73493976	79
HIF1A	Hypoxia-inducible factor 1-alpha	0.01139618	0.73493976	79
EGF	Proepidermal growth factor	0.01558726	0.73053892	78
MYC	Myc proto-oncogene protein	0.01512287	0.72189349	77
EGFR	Epidermal growth factor receptor	0.02954812	0.72189349	76
MMP9	Matrix metalloproteinase-9	0.02061406	0.72619048	76
PPARG	Peroxisome proliferator-activated receptor gamma	0.01436399	0.71764706	74

**Table 3:** Molecular docking results.

Active ingredient	Binding energy (kcal·mol^−1^)				
	AKT1	TNF	IL6	TP53	VEGFA
Quercetin	−4.49	−5.13	−4.9	−5.52	−4.23
Luteolin	−6.21	−7.4	−4.48	−6.07	−5.41
Tanshinone IIA	−7.97	−9.42	−6.84	−9.07	−7.73
*β*-Sitosterol	−7.99	−9.30	−6.8	−9.27	−6.50
Dihydrotanshinlactone	−6.07	−6.82	−5.48	−7.2	−6.13

## Data Availability

The data used to support this study are available from the corresponding author upon request.

## References

[1] Li H., Hu L., Zheng Y. (2021). Analysis of the epidemiological burden of psoriasis in China based on the big data of global burden of disease study(GBD). *The Chinese Journal of Dermatovenereology*.

[2] Hu J. Z., Billings S. D., Yan D., Fernandez A. P. (2020). Histologic comparison of tumor necrosis factor-*α* inhibitor-induced psoriasis and psoriasis vulgaris. *Journal of the American Academy of Dermatology*.

[3] Cao Y., Zhu K., Pan F. (2020). Modern literature study on syndrome distribution and Chinese medicine medication regularity in treatment of psoriasis vulgaris. *Journal of Guangzhou University of Traditional Chinese Medicine*.

[4] Ji Y., Li W., Lin Y., Shuchan Z., Xuan L. (2022). Discussion on the treatment of plaque psoriasis with fire acupuncture combined with cotton moxibustion based on collateral disease theory. *Journal of Liaoning University of Traditional Chinese Medicine*.

[5] Guo J., Guo W., Li H. (2012). Clinical observation on Treating 30 cases of psoriasis vulgaris from collateral disease. *Guiding Journal of Traditional Chinese Medicine and Pharmacy*.

[6] Ru J., Li P., Wang J. (2014). TCMSP: a database of systems pharmacology for drug discovery from herbal medicines. *Journal of Cheminformatics*.

[7] Bateman A., Martin M., Orchard S. (2021). UniProt: the universal protein knowledgebase in 2021. *Nucleic Acids Research*.

[8] Stelzer G., Rosen N., Plaschkes I. (2016). The GeneCards suite: from gene data mining to disease genome sequence analyses. *Current Protocols in Bioinformatics*.

[9] Hamosh A., Amberger J. S., Bocchini C., Scott A. F., Rasmussen S. A. (2021). Online mendelian inheritance in man (OMIM): victor McKusick’s magnum opus. *American Journal of Medical Genetics, Part A*.

[10] Wang Y., Zhang S., Li F. (2019). Therapeutic target database 2020: enriched resource for facilitating research and early development of targeted therapeutics. *Nucleic Acids Research*.

[11] Piñero J., Ramírez-Anguita J. M., Saüch-Pitarch J. (2019). The DisGeNET knowledge platform for disease genomics: 2019 update. *Nucleic Acids Research*.

[12] Wishart D. S., Feunang Y. D., Guo A. C. (2018). DrugBank 5.0: a major update to the DrugBank database for 2018. *Nucleic Acids Research*.

[13] Ragueneau E., Shrivastava A., Morris J. H., del-Toro N., Hermjakob H., Porras P. (2021). IntAct App: a Cytoscape application for molecular interaction network visualization and analysis. *Bioinformatics*.

[14] Szklarczyk D., Gable A. L., Nastou K. C. (2021). The STRING database in 2021: customizable protein-protein networks, and functional characterization of user-uploaded gene/measurement sets. *Nucleic Acids Research*.

[15] Huang D. W., Sherman B. T., Lempicki R. A. (2009). Systematic and integrative analysis of large gene lists using DAVID bioinformatics resources. *Nature Protocols*.

[16] Berman H. M., Westbrook J., Feng Z. (2000). The protein data bank. *Nucleic Acids Research*.

[17] Hsin K.-Y., Ghosh S., Kitano H. (2013). Combining machine learning systems and multiple docking simulation packages to improve docking prediction reliability for network pharmacology. *PLoS One*.

[18] Mz B. H. (2020). Shortcuts for faster image creation in PyMOL. *Protein Science: A Publication of the Protein Society*.

[19] Trott O., Olson A. J. (2010). AutoDock Vina: improving the speed and accuracy of docking with a new scoring function, efficient optimization, and multithreading. *Journal of Computational Chemistry*.

[20] Dong X., Luo S., Bai Y. (2021). Study on current situation of TCM treatment for psoriasis. *Beijing Journal of Traditional Chinese Medicine*.

[21] Fei W., Tang H., Yang S., Venereol C. J. D. (2018). Changes of cutaneous microcirculatory in psoriasis. *The Chinese Journal of Dermatovenereology*.

[22] Jia J., Mo X., Liu J. (2020). Mechanism of danshensu-induced inhibition of abnormal epidermal proliferation in psoriasis. *European Journal of Pharmacology*.

[23] Jia J., Li C., Yang J. (2018). Yes-associated protein promotes the abnormal proliferation of psoriatic keratinocytes via an amphiregulin dependent pathway. *Scientific Reports*.

[24] Wang S., Zhu L., Xu Y., Qin Z., Xu A. (2020). Salvianolic acid B ameliorates psoriatic changes in imiquimod-induced psoriasis on BALB/c mice by inhibiting inflammatory and keratin markers via altering phosphatidylinositol-3-kinase/protein kinase B signaling pathway. *Korean Journal of Physiology and Pharmacology*.

[25] Jing H., Zhou H., Duan D. (2013). The influence of angelica polysaccharide on the expression of NF-*κ*B and IFN-*γ* in co-culture of peripheral blood mononuclear cells and keratinocytes from psoriatic patients. *China Journal of Leprosy and Skin Diseases*.

[26] Efferth T., Oesch F. (2020). Anti-inflammatory and Anti-cancer Activities of Frankincense: Targets, Treatments and toxicities. *Seminars in Cancer Biology*.

[27] Maramaldi G., Togni S., Di Pierro F., Biondi M. (2014). A cosmeceutical formulation based on boswellic acids for the treatment of erythematous eczema and psoriasis. *Clinical, Cosmetic and Investigational Dermatology*.

[28] Shen Y. (2020). *The Role and Mechanisms of Guggulsterone in Keratinocytes[D]*.

[29] Chen H., Lu C., Liu H. (2017). Quercetin ameliorates imiquimod-induced psoriasis-like skin inflammation in mice via the NF-*κ*B pathway. *International Immunopharmacology*.

[30] Zhou W., Hu M., Zang X. (2020). Luteolin attenuates imiquimod-induced psoriasis-like skin lesions in BALB/c mice via suppression of inflammation response. *Biomedicine & Pharmacotherapy*.

[31] Weng Z., Patel A. B., Vasiadi M., Therianou A., Theoharides T. C. (2014). Luteolin inhibits human keratinocyte activation and decreases NF-*κ*B induction that is increased in psoriatic skin. *PLoS One*.

[32] Sundarrajan S., Nandakumar M. P., Prabhu D., Jeyaraman J., Arumugam M. (2020). Conformational insights into the inhibitory mechanism of phyto-compounds against Src kinase family members implicated in psoriasis. *Journal of Biomolecular Structure and Dynamics*.

[33] Lv J., Zhou D., Wang Y. (2020). Effects of luteolin on treatment of psoriasis by repressing HSP90. *International Immunopharmacology*.

[34] Li F., Xu R., Zeng Q. (2012). Tanshinone IIA inhibits growth of keratinocytes through cell cycle arrest and apoptosis: underlying treatment mechanism of psoriasis. *Evidence-based complementary and alternative medicine*.

[35] Kuai L., Luo Y., Qu K. (2021). Transcriptomic analysis of the mechanisms for alleviating psoriatic dermatitis using taodan granules in an imiquimod-induced psoriasis-like mouse model. *Frontiers in Pharmacology*.

[36] Tang L., He S., Wang X. (2018). Cryptotanshinonereduces psoriatic epidermal hyperplasia via inhibiting the activation of STAT3. *Experimental Dermatology*.

[37] Yang X., Wang X., Zhang X. (2021). Role of Akt in epidermal proliferation in psoriasis vulgaris. *Chinese Journal of Dermatovenereology of Integrated Traditional and Western Medicine*.

[38] Duan Q., Wang G., Wang M. (2020). LncRNA RP6‐65G23.1 accelerates proliferation and inhibits apoptosis via p‐ERK1/2/p‐AKT signaling pathway on keratinocytes. *Journal of Cellular Biochemistry*.

[39] Li B., Lei J., Yang L. (2019). Dysregulation of akt-FOXO1 pathway leads to dysfunction of regulatory T cells in patients with psoriasis. *Journal of Investigative Dermatology*.

[40] Armstrong A. W., Read C. (2020). Pathophysiology, clinical presentation, and treatment of psoriasis. *JAMA*.

[41] Luengas‐Martinez A., Hardman‐Smart J., Paus R., Young H. S. (2020). Vascular endothelial growth factor‐A as a promising therapeutic target for the management of psoriasis. *Experimental Dermatology*.

[42] Benhadou F., Glitzner E., Brisebarre A. (2020). Epidermal autonomous VEGFA/Flt1/Nrp1 functions mediate psoriasis-like disease. *Science Advances*.

[43] Ehst B., Wang Z., Leitenberger J. (2021). Synergistic induction of IL-23 by TNF*α*, IL-17A, and EGF in keratinocytes. *Cytokine*.

[44] Wang W., He Y., Xu H. (2020). Update of epidermal growth factor receptor signaling pathway in the pathogenesis of related skin diseases. *China Journal of Leprosy and Skin Diseases*.

[45] Sa G., Das T. (2008). Anti cancer effects of curcumin: cycle of life and death. *Cell Division*.

[46] Moorchung N., Vasudevan B., Dinesh Kumar S., Muralidhar A. (2015). Expression of apoptosis regulating proteins p53 and bcl-2 in psoriasis. *Indian Journal of Pathology & Microbiology*.

[47] Zhang D., Chen Y., Chen L. (2016). Ultraviolet irradiation promotesFOXP3transcription via p53 in psoriasis. *Experimental Dermatology*.

[48] Wei Z., Li T., Sun Y. (2021). Daturataturin A, a withanolide in Datura metel L., induces HaCaT autophagy through the PI3K‐Akt‐mTOR signaling pathway. *Phytotherapy Research*.

[49] Bebars S. M. M., Al-Sharaky D. R., Gaber M. A., Afify D. R. (2017). Immunohistochemical expression of caspase-3 in psoriasis. *Journal of Clinical and Diagnostic Research: Journal of Clinical and Diagnostic Research*.

[50] Morelli M., Scarponi C., Mercurio L. (2018). Selective immunomodulation of inflammatory pathways in keratinocytes by the janus kinase (JAK) inhibitor tofacitinib: implications for the employment of JAK-targeting drugs in psoriasis. *Journal of immunology research*.

[51] Xu F., Xu J., Xiong X., Deng Y. (2019). Salidroside inhibits MAPK, NF-*κ*B, and STAT3 pathways in psoriasis-associated oxidative stress via SIRT1 activation. *Redox Report*.

[52] Yang Z., Chen Z., Wang C., Huang P., Luo M., Zhou R. (2021). STAT3/SH3PXD2A-AS1/miR-125b/STAT3 positive feedback loop affects psoriasis pathogenesis via regulating human keratinocyte proliferation. *Cytokine*.

[53] Abdou A. G., Farag A. G. A., Hammam M., Taie D. M., Abdelaziz R. A. (2018). Immunohistochemical expression HIF1*α* in chronic plaque psoriasis, an association with angiogenesis and proliferation. *Journal of Immunoassay and Immunochemistry*.

[54] Blunder S., Krimbacher T., Moosbrugger‐Martinz V., Gruber R., Schmuth M., Dubrac S. (2021). Keratinocyte‐derived IL‐1*β* induces PPARG downregulation and PPARD upregulation in human reconstructed epidermis following barrier impairment. *Experimental Dermatology*.

[55] Amezcua-Guerra L. M., Bojalil R., Espinoza-Hernandez J. (2018). Serum of patients with psoriasis modulates the production of MMP-9 and TIMP-1 in cells of monocytic lineage. *Immunological Investigations*.

[56] Cibrian D., de la Fuente H., Sánchez-Madrid F. (2020). Metabolic pathways that control skin homeostasis and inflammation. *Trends in Molecular Medicine*.

[57] Trafford A. M., Parisi R., Kontopantelis E., Griffiths C. E. M., Ashcroft D. M. (2019). Association of psoriasis with the risk of developing or dying of cancer. *JAMA Dermatology*.

[58] Vaengebjerg S., Skov L., Egeberg A., Loft N. D. (2020). Prevalence, incidence, and risk of cancer in patients with psoriasis and psoriatic arthritis. *JAMA Dermatology*.

[59] Prizment A. E., Alonso A., Folsom A. R. (2011). Association between psoriasis and incident cancer: the Iowa’s Women’s Health Study. *Cancer Causes & Control*.

[60] Chiesa Fuxench Z. C., Shin D. B., Ogdie Beatty A., Gelfand J. M. (2016). The risk of cancer in patients with psoriasis. *JAMA Dermatology*.

[61] Furue K., Ito T., Tsuji G., Kadono T., Furue M. (2019). Psoriasis and the TNF/IL23/IL17 axis. *Societa italiana di dermatologia e sifilografia*.

[62] Zhu W.-J., Li P., Wang L., Xu Y.-C. (2020). Hypoxia-inducible factor-1: a potential pharmacological target to manage psoriasis. *International Immunopharmacology*.

[63] Tang W., Long T., Li F. (2021). HIF-1*α* may promote glycolysis in psoriasis vulgaris via upregulation of CD147 and GLUT1. *Journal of Central South University*.

[64] Zhou F., Zhu Z., Gao J. (2018). NFKB1 mediates Th1/Th17 activation in the pathogenesis of psoriasis. *Cellular Immunology*.

